# Circulating biosignatures in multiple myeloma and their role in multidrug resistance

**DOI:** 10.1186/s12943-022-01683-w

**Published:** 2023-04-29

**Authors:** S. Rajeev Krishnan, M. Bebawy

**Affiliations:** Sydney, NSW 200 Australia

**Keywords:** Biomarkers, Extracellular vesicles, Liquid biopsy, Microparticles, Multidrug resistance multiple myeloma, Personalized medicine

## Abstract

A major obstacle to chemotherapeutic success in cancer treatment is the development of drug resistance. This occurs when a tumour fails to reduce in size after treatment or when there is clinical relapse after an initial positive response to treatment. A unique and serious type of resistance is multidrug resistance (MDR). MDR causes the simultaneous cross resistance to unrelated drugs used in chemotherapy. MDR can be acquired through genetic alterations following drug exposure, or as discovered by us, through alternative pathways mediated by the transfer of functional MDR proteins and nucleic acids by extracellular vesicles (M Bebawy V Combes E Lee R Jaiswal J Gong A Bonhoure GE Grau, 23 9 1643 1649, 2009).

Multiple myeloma is an incurable cancer of bone marrow plasma cells. Treatment involves high dose combination chemotherapy and patient response is unpredictable and variable due to the presence of multisite clonal tumour infiltrates. This clonal heterogeneity can contribute to the development of MDR. There is currently no approved clinical test for the minimally invasive testing of MDR in myeloma.

Extracellular vesicles comprise a group of heterogeneous cell-derived membranous structures which include; exosomes, microparticles (microvesicles), migrasomes and apoptotic bodies. Extracellular vesicles serve an important role in cellular communication through the intercellular transfer of cellular protein, nucleic acid and lipid cargo. Of these, microparticles (MPs) originate from the cell plasma membrane and vary in size from 0.1-1um. We have previously shown that MPs confer MDR through the transfer of resistance proteins and nucleic acids. A test for the early detection of MDR would benefit clinical decision making, improve survival and support rational drug use. This review focuses on microparticles as novel clinical biomarkers for the detection of MDR in Myeloma and discusses their role in the therapeutic management of the disease.

## Background

### The problem of multiple myeloma

Myeloma is a plasma cell (PC) cancer and comprises the second most common hematological cancer with approx. 160,000 global cases [15, 120]. Worldwide, cases have increased by 126% since 1990 with Australasia having the highest age-standardised incidence and death-rate followed by North America and Europe [26]. In Australia, myeloma has an estimated mean yearly cost per treatment per patient of approximately $25,000 [125]. Despite significant improvements in the 5-year survival rate (43% in the period of 2006–2010), the outcomes for older patients remain poor with a 19% 5-year survival rate in individuals aged 80-years or over [71]. A global increased incidence has been reported particularly in men, people aged 50 years or older from developed countries [59].

Myeloma is characterized by multi-site tumor infiltrates of aberrant plasmacytomas, which are predominantly present throughout the axial skeleton. The presence of clonal heterogeneity among these, contribute to the development of resistant clones and variability in disease and in patient outcomes [4, 22, 51, 89]{Pinto, 2020 #237.

### MDR in multiple myeloma

In Australia, the five year survival for myeloma is 51%, an improvement from the 28% rate of the 1990s [1]. In 2020, approximately, 2400 new myeloma cases were reported in Australia{AIHW, 2020 #223}. The inclusion of proteasome inhibitors and immunomodulatory agents have contributed to these improvements. Despite these clinical benefits, therapeutic success remains compromised by the development of MDR. MDR in myeloma is associated with the overexpression of P-gycoprotein (P-gp) Abbaszadegan et al. [2, 96] It was shown in the 90 s that at least 5% of untreated myeloma cases present with high P-gp levels limiting the success of induction therapy. Furthermore, 33% of patients at relapse are positive for P-gp [35]. The relevance of ABC- transporter mediated multidrug resistance is a point of discussion in current times as well with access to novel therapies {Besse, 2018 #220}{Gozzetti, 2022 #219}{Mynott, 2021 #222}{Uckun, 2022 #217}. Earlier strategies to counteract MDR included the addition of inhibitors to treatment combinations, but these were of limited success, because of dose-limitations and altered pharmacokinetic profiles [12, 20, 38, 94, 122, 134].

Adding to this, the myeloma ‘side population’ expressing P-gp, is implicated in relapse in myeloma [4, 84, 89, 102]. At diagnosis, myeloma patients can present with subclones of aberrant plasma cells{Corre, 2015 #238}, commonly referred to as *‘side populations’{Loh, 2008 #171}{Agarwal, 2010 #157;Matsui, 2008 #170}*. The side population were first identified by their ability to efflux Hoechst 33,342 in a unique pattern and they typically lack CD138 on their surface{Goodell, 1996 #239}{Loh, 2008 #171} The side population cells have ‘*stem cell like characteristics’, possess self-renewal characteristics, lack CD138 (plasma cell marker)* and overexpress multidrug resistance proteins such as P-gp [7, 54]{Katz, 2008 #241}.

### Current clinical approaches and their limitations

Many diagnostic tests are used to support the clinical management of myeloma [70], however have limitations. Conventional serological markers (i.e. serum free-light chains and monoclonal paraprotein) are limited to single analytes and do not provide information on molecular markers of disease progression or the detection of resistance mechanisms (Table 1). These markers provide essential biochemical measures of stage and disease progression, renal function, tumor burden, bone physiology, therapeutic outcome and the presence of inflammation commonly associated with malignancy as summarised in the following paragraphs (D. E. Joshua, Brown, & Gibson, 1994).

**Table 1:** Systemic biomarkers in MM

Systemic Markers	Clinical indication	Application
M Protein	Tumour burden	Staging
Light chains	Tumour burden	Staging
Beta 2 microglobulin	Tumour burden	Staging
Serum CD138, circulating plasma cells, cell free DNA	Tumour burden/minimal residual disease (MRD)	Staging and prognosis
C Reactive Protein, interlukin 6 and receptor (IL-6 and ILR)	Inflammation	Disease progression,
ThymidineKinase/Lactate dehydrogenase	Tumour burden	Prognosis
Bone markers (PICP, ICTP)	Bone Physiology	Diagnosis and Prognosis, disease progression
Serum Creatinine	Renal function	Diagnosis and Prognosis, disease progression

### A. Markers of cellular change

***Thymidine kinase (TK)*** is a phospho-transferase involved in the DNA salvage pathway and specifically catalyses phosphorylation of deoxythymidine. Serum levels of thymidine kinase (TK) are an indirect measure of plasma cell proliferation [17, 88]. Numerous retrospective studies support the prognostic significance of systemic TK levels in MM patients [8, 16, 88] R. Brown et al., observed that serum TK levels > 11 U/l as associated with shorter survival rates indicating its significance as a marker of tumor endurance. However, the predictive power of serum TK levels is dependent on the regimens used. It provides significant prognosis with single agent melphalan although, is not useful in combination therapy [17].

***β-2-microglobulin (β***_*2*_*M)* is a low molecular weight cytoplasmic membrane protein expressed on the surface of all nucleated cells except for red blood cells. It is shed systemically post cell death or following membrane remodelling [53]. It is a 12 kDa surface protein closely associated with major histocompatibility complex class 1 heavy chain and is a member of the immunoglobulin gene superfamily [101, 103]. An elevated level of β_2_M is one of the most significant prognostic measures in MM at diagnosis [27]. β_2_M levels indicate tumor burden and renal involvement. The current International Staging System (ISS) uses serum levels of β_2_M and serum albumin for staging and risk-stratification together with patient genotype [51]. β2M is only useful in the case of symptomatic myeloma and cannot be used to gauge the transition between benign monoclonal gammopathy of undetermined significance (MGUS), asymptomatic smoldering myeloma and multiple myeloma [79]. β_2_M can be a misleading marker of tumor load when it comes to individual patients. A recent study showed that where 5 out of 6 patients with stage II myeloma and 5 of 11 patients with stage III myeloma showed normal β_2_M levels. This means 58.8% of patients with substantial infiltration in the bone marrow showed false-negative β2-microglobulin levels [28].

#### Paraprotein or monoclonal (Mprotein)

Systemic Mprotein is a hallmark of secretory myeloma and severely impairs immune capacity of patients as a consequence of its clonal incompetence [80]. Paraprotein levels are routinely monitored in secretory MM with an elevated level indicating progressive disease [108]. Although, Mprotein (> 3 g/dl) in blood is used to differentiate between MGUS and myeloma, quantitative levels of Mprotein are not an exclusive marker in myeloma [115].

#### Free light chains

The ratio of free light chains (κ/λ) (0.26–1.65 mg/dL) of the monoclonal immunoglobulin is a reliable prognostic indicator in MM especially in non-secretory myeloma, where the classic M protein secretion is lacking and is an indirect measure of clonality. Nevertheless, serum free light chain assays have limitations including sample dilution anomalies, calibration problems and limits of detection, which may result in erroneous inference of clinical significance [124].

#### Acute phase proteins

An acute phase response is typically associated with myeloma [5, 57]. Interleukin -6 (IL-6) has a significant role in B cell differentiation; especially in the final differentiation of B cells to mature plasma cells. A number of studies have indicated that targeting the IL-6 pathway inhibits myeloma growth through inhibition of the nuclear factor kappa B and/or Janus kinase signalling pathways [56, 75]. IL-6 also protects myeloma cells against dexamethasone-induced apoptosis by activating protein tyrosine phosphatase [57]. Together with IL-6, IL-2, IL-1β and soluble IL-6 receptor (S-IL-6R) have also been shown to affect the survival of myeloma patients [90, 106]. Stromal cell derived factor (SDF)-1 upregulates IL-6 secretion in myeloma, which ensures tumor growth, survival, and migration [56]. Likewise, glycoprotein-130 (gp130), a subunit of IL-6 receptor family, is also present in its soluble effector form in circulation. This inhibits the growth of MM cells through its association with IL-6 and s-IL6-R. Thus, a ratio of s-IL6-R to gp130 is of prognostic significance [106]. IL-6 also plays a major role in bone resorption by myeloma cells. IL-6 activates osteoclasts and promotes defective bone physiology thus provide a measure of the extent of bone disease in myeloma patients. C-Reactive Protein (CRP), an inflammatory marker is also regulated by IL-6 and provides an indirect marker of IL-6 levels and disease burden. A disadvantage of IL-6 as a systemic marker of myeloma is its ubiquitous nature with respect to inflammation generally and is not specific to myeloma [72].

### B. Markers of aberrant cellular metabolism

Typical of malignant cells, myeloma cells are characterized by aberrant glycolysis [34]. Elevated levels of lactate dehydrogenase (LDH), an enzyme involved in anaerobic cellular metabolism is a prognostic measure in MM [34]. High levels of LDH (≥ 300 IU/L) have been shown to correlate with lower overall survival and failure to respond to conventional MM therapy (67). Dimopoulos et al. [34] showed that only 20% of patients with high LDH levels responded to treatment compared to 57% patients with low LDH levels [34]. However, LDH’s potential to help in risk-stratification of patients with respect to novel agents including immunomodulators and proteasome inhibitors remains to be investigated.

### C. Markers of altered skeletal physiology

Myeloma is typically associated with defective bone physiology. This is clinically monitored using systemic markers of bone formation and bone degradation. The rate of bone formation is measured indirectly with serum alkaline phosphatase and PICP (type 1 carboxy terminal propeptide, type 1 collagen biosynthesis marker). Similarly, bone resorption is marked by collagen breakdown products such as ICTP (type 1 carboxy terminal cross-linked telopeptide, > 5.0 µg/l) [3, 29, 36]. PICP and ICTP levels; are only indicative of abnormal bone physiology in myeloma along with the help of imaging techniques.

### D. Markers of systemic nucleic acids

In the case of extram-edullary myeloma, time-matched extramedullary plasmacytoma biopsies, bone marrow aspirates, and plasma samples from 8 patients were analysed and Zhou et al. found that circulating tumor DNA is an appropriate substitute for extramedullary plasmacytoma biopsy for genomic profiling and prognostic assessment{Zou, 2005 #93}. However, currently the method to implement this is with the use of Next-generation sequencing (NGS). NGS is associated with a number of technical challenges which limit its routine use clinically including; sample sufficiency as well low sensitivity in identifying structural and copy number variations {Ulahannan, 2013 #226}. It has been suggested that NGS should only be ordered as a second-tier test for high-risk patients{Mauer, 2014 #227}.

### E. Markers of Minimal Residual Disease (MRD)

Minimal residual disease monitoring is an important prognostic marker [40, 98, 111]. Bone marrow biopsy is the gold-standard approach used, however this is invasive, prevents routine use and is limited by sample bias. The recent FDA approved ClonoSEQ assay is limited for the same reasons. Multi-parameter flow cytometric immunophenotypic analysis of BM aspirate /biopsy is a valuable approach used in myeloma, however it is also dependent on sampling and often is limited by loss of sample during preparation [23, 67].

ClonoSEQ assay is an in vitro diagnostic test from Adaptive Biotechnologies; Seattle WA. The assay uses multiplex polymerase chain reaction and next generation sequencing to identify and quantify disease-associated sequence rearrangements (or clonotypes) of the IgH, IgK, and IgL receptor genes, as well as IgH/BCL1 and IgH/BCL2 translocations, in DNA extracted from bone marrow [24]. This assay is optimised to gauge the minimal residual disease [68], however, rely on invasive bone marrow sampling. Other limitations of the assay include its reliance on high disease burden in order to identify disease-associated clonotypes {Ching, 2020 #55}. ClonoSEQ depends on both the volume and the cellularity of bone marrow sample input and the limit of detection has slight upward bias that may cause MRD frequencies to be overestimated [24]. Besides, the assay requires a baseline patient sample for identification and detection of immunoglobulin gene re-arrangements {Bal, 2020 #228}.

There is currently no test available for the minimally invasive testing of MDR in myeloma. An ideal test would (i) directly measure markers of MDR during routine follow up*,* (ii) be minimally-invasive, (iii) be representative of multi-site infiltrates and (iv) allow for simultaneous analysis of disease burden. Such a test would support timely strategies and rational use of costly antibodies, emerging cell therapies in response to MDR, reducing relapse and improving survival. It is pertinent to note that any new paradigms designed to counteract drug resistance in myeloma have been met with inherent and acquired resistance mechanisms{Uckun, 2022 #217}.

As myeloma constantly changes in response to treatment, it is important to have a test which can be used to support drug selection in response to changing disease, development of MDR and evolving putative drug targets.

### Extracellular vesicles and MDR

EVs are broadly classified into exosomes, microvesicles and apototic bodies based on their size and origin [19], Minciacchi, Freeman, & Di Vizio, 2015). EVs are mediators of intercellular communication. Microvesicles/microparticles are formed by the outward budding and fission of the plasma membrane while exosomes are formed within the endosomal network and released upon fusion of multi-vesicular bodies with the plasma membrane. The larger of the three—apoptotic bodies are released as blebs of cells undergoing apoptosis{Yáñez-Mó, 2015 #229}. EVs, contain cargo such as nucleic acids, proteins, lipids and metabolites from the originating cell and they transfer cellular cargo between cells. MPs present as ideal biomarker candidates due to their larger size which makes them readily detectable using routine flow cytometry especially in a hematological clinical setting.

**Fig. 1:**
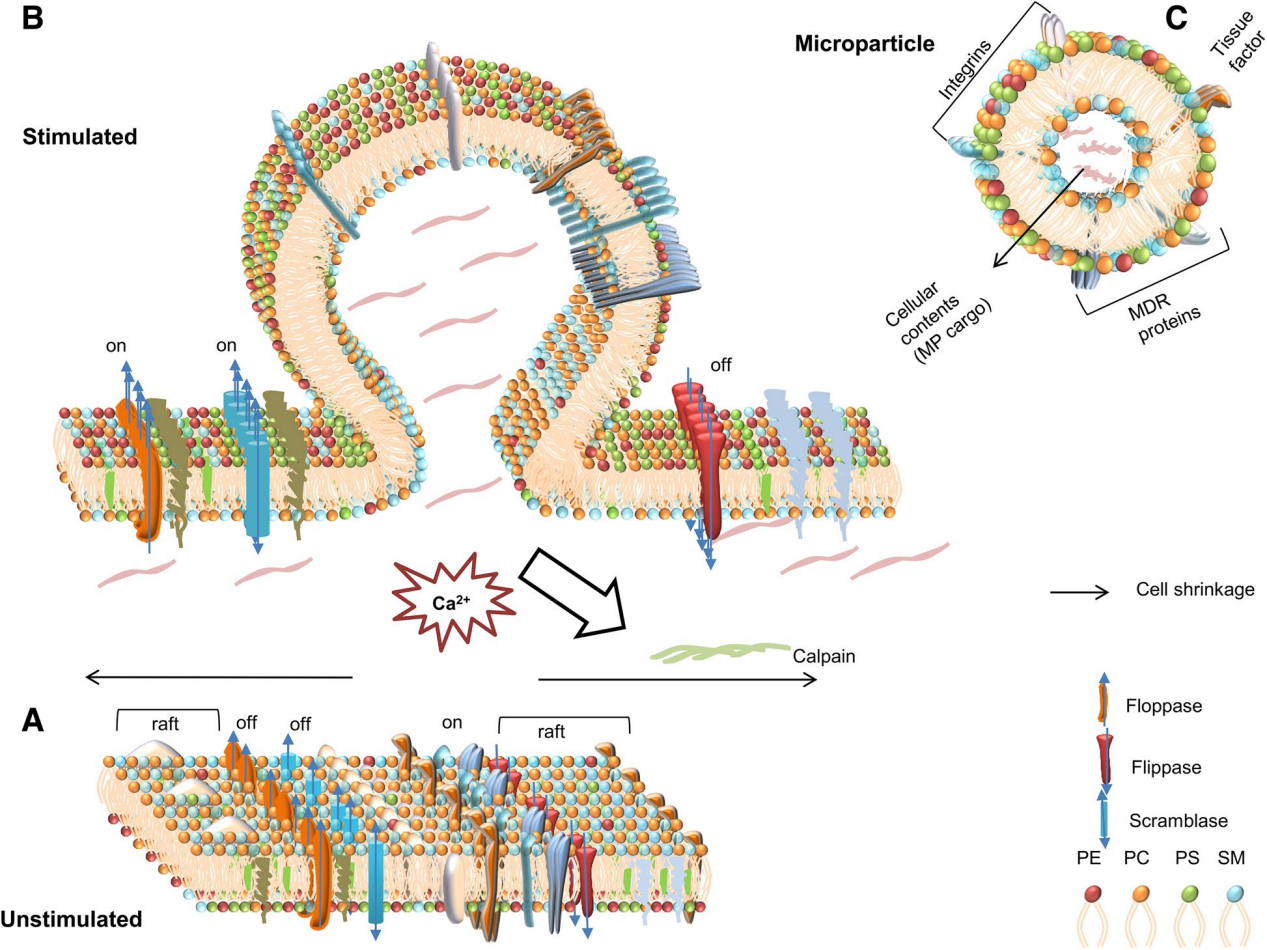
Schematic representation of microparticle physiology. **A.** Membrane asymmetry in an unstimulated cell: The plasma membrane lipid bilayer harbors, transmembrane proteins, integrins, and ion channels. The membrane proteins are segregated laterally in lipid rafts. In the unstimulated state, the outer layer of plasma membrane is enriched with phosphatidyl choline (PC) and sphingomyelin (SM) whereas the inner layer is enriched by phosphatidyl ethanolamine (PE) and phosphatidyl serine (PS). The inward directed phosholipid pump flippase is active at this stage and maintains membrane asymmetry of the lipid bilayer. **B.** Disruption of cytoskeleton and plasma membrane vesiculation. Membrane vesiculation occurs as a response to various stimuli and as a result of cytoskeletal disruption during membrane remodelling. An influx of calcium ions in the cytosol activates cytosolic enzyme calpain disrupting the cytoskeleton, switches on floppase (outward phospholipid pump) and scramblase disrupting the (bidirectional) on the membrane. Floppase redirect PS to the outer leaflet causing exposure of PS—a hallmark of MPs. MPs package cytoskeletal elements, nucleic acids, proteins, lipids and carry the surface markers from the cell of origin resulting in cell shrinkage. Segregation of lipids and proteins into lipid rafts enable specific packaging of contents while membrane vesiculation resulting in diversity in MPs even if the origin is same.** C.** Diagrammatic representation of a microparticle. Microparticles (MPs) on the surface carries the hallmark of their cells of origin, membrane transporters, tissue factors and exposed phosphatidyl serine (PS). On the inside, MPs harbour selectively packaged cellular contents such as bioactive proteins, lipids and nucleic acids

MPs are shed spontaneously in blood from the surface of cells and range in size from 0.1-1um (Fig. 1) [46]. They carry proteins, lipids and nucleic acids from the originating/parent cells [65] MPs are part of normal cell biology and as a result, MPs are detected systemically in healthy individuals; nonetheless, higher levels are indicative of cellular activation across several pathologies [37, 44, 127, 133]{Li, 2021 #231}{Zahran, 2021 #232}. MPs have been implicated in vascular biology, inflammatory disease states such as cerebral malaria, and are known to transfer deleterious traits such as multidrug resistance, metastatic capacity, and immune evasion in cancer [10, 41, 100]. Disease states such as cancer, vasculitis, arthritis, autoimmune disorders and AIDS are associated with elevated MP numbers in circulation relative to healthy subjects. The detection of circulating cancer-derived MPs from different cancers, has defined them as promising “surrogate” markers in compartmentalised malignancies (i.e. brain and bone) [12, 30, 40, 45, 66].

### Extracellular vesicles and cancer

Cancer derived vesicles (specifically microparticles) were first described by Friend C et al. in 1978 as “rare pleomorphic membrane line particles ranging broadly in size between 400 and 1200 A^0^” [42]. Cancer MPs were later shown to be associated with hypercoagulation associated with cancer, due to the presence of phosphatidylserine and tissue factor on the surface of cancer derived MPs [6, 21].

Since these early studies, cancer derived MPs have been shown to play an important role in metastasis, angiogenesis, immune evasion, drug sequestration{Jaiswal, 2014 #57}. MP s are of diagnostic and prognostic potential in breast cancer {Li, 2021 #231;Pokharel, 2016 #117;Pokharel, 2014 #88;Toth, 2008 #70;Yang, 2011 #95}. diabetes {Tramontano, 2010 #142}glioma{Li, 2013 #87}acute myeloid leukemia {Kalinkovich, 2006 #234}pancreatic cancer{Woei-A-Jin, 2016 #235}prostate cancer {Rogers, 2020 #236}and many more chronic diseases.

In the context of MDR, the regulatory control of MDR was always known to occur via pre or post transcriptional mechanisms regulating MDR transporter expression {Jaiswal, 2014 #233}. However, Bebawy et al., discovered that MDR could also be acquired through intercellular transfer of resistance proteins and nucleic acids by extracellular vesicles, specifically, microparticles (MP) [10]{Jaiswal, 2012 #85}{Gong, 2014 #86;Jaiswal, 2012 #73}; and their presence in blood constitute part of the *tumor circulome,* making them ideal blood biomarker candidates in myeloma [31]. MPs are a vehicle for the transfer of resistance proteins such as P-gp and multidrug resistance associated protein -1 (MRP1) between cells [10, 65, 86, 87]{Gong, 2014 #86}.

MPs shed from malignant cells have the capacity to ensure the transfer of deleterious cancer cell traits to recipient cells leading to trait dominance within the recipient cell population [48, 62, 86]. MPs achieve this through intercellular transfer of cargo but also through a remarkable capacity to “re-template” the transcriptional and protein landscape of cells [48, 62, 86]. MPs induce a rapid transcriptional response in recipient cells which has been shown to be induced through selective packaging of unique RNA species in MP cargo [82]. Overall, MPs support a complex cancer survival phenotype, associated with the transfer of MDR, biophysical alterations, enhanced metastasis, drug sequestration and immune evasion [49, 63, 105].

### Extracellular vesicles and myeloma

Extracellular vesicles are valuable biomarkers in ‘cancer liquid biopsy’ [30, 31, 107]. The consideration of MP counts and molecular profile has been shown to correspond to disease pathology and/or treatment sensitivity at an individual level [76, 77, 107, 126, 127].

There is increasing evidence on the role these sub-micron particles playing on various pathophysiologies including myeloma [31, 76, 77, 91, 107, 112, 132, 135].

Benameur et al. [11], previously showed the presence of MPs in myeloma in the 5T2MM myeloma mouse model. This study demonstrated that elevated levels of MPs were detected in late stage disease relative to early stage [11]. This study was focussed on a C57BL/KaLwRij mouse model of myeloma, whereby CD138^+^ MPs were enumerated in early stage disease and compared to that of late stage disease [11]. The circulating MP number and phenotypic subtypes such as endothelial, platelet, procoagulant, RBC and WBC derived MP levels in the late-stage myeloma (10–12 wks) were significantly higher compared to the control cohort. However, the MP count in the early stage of MM (6 wks) was significantly lower than the control groups across all the sub-types. MP count in the bone marrow was also elevated in late-stage disease and expressed CD138^+^ as demonstrated by transmission electron microscopy.

Increased risk of venous thromboembolism is a complication associated with myeloma pathology and it manifests in the initial induction therapy phase for many patients [6]. MP associated tissue factor activity is commonly observed in cancer and in myeloma, MPs also support hypercoagulable state and tissue factor bearing MPs can interact with activated platelets [6, 32]. Platelet MPs and their clinical role in thromboembolic risk have been studied with the introduction of IMiDs in the treatment of myeloma [25]. Auwerda et al. reported the higher incidence of platelet derived MP-tissue factor activity in de novo patients to that of healthy volunteers [6]. The study showed that in myeloma, tissue factor activity associated with platelet derived MPs was higher in de novo cohorts than healthy volunteers. The platelet MP count was also shown to decline in response to treatment in myeloma patients [6].

MPs secreted from the human myeloma cell line RPMI8226 and in vivo have also been shown to promote angiogenesis through the transfer of oncogenic CD138 to endothelial cells [83]. The transfer of CD138 augmented the secretion of angiogenic regulators (vascular endothelial factor (VEGF) and IL-6 by recipient cells, resulting in increased proliferation, invasion and formation of tubes in vitro and in vivo. This study demonstrates the significant pathophysiological role of CD138 enriched MPs as mediators of new vasculature formation aiding in pathophysiological dissemination of myeloma [83].

Multiple myeloma is associated with complex network of mediators within the bone marrow microenvironment. Roccaro et al. established that bone marrow-mesenchymal stromal cells (BMSCs) release vesicles (exosomes 50–100 nm size) and that the cargo of of MM BM-MSCs differed in content and function to those derived from healthy cells [112]. The study demonstrated PKH67 labelled healthy, and myeloma BM-MSC-derived exosomes were readily taken up by myeloma cells. BM-MSC-derived exosomes from myeloma patients induced tumor growth in vivo and aided in dissemination of tumor cells in an in vivo translational model of myeloma. The study further analysed the proteomic content of the normal and MM BM-MSC-derived exosomes to elucidate the tumor-initiating capacity of MM-BMSC derived exosomes. The tumor suppressor gene regulator *miRNA- miR-15a* was down-regulated in myeloma BM-MSC derived exosomes compared to normal BM-MSC derived exosomes indicative of a tumor-suppressive role of mesenchymal stroma cell derived exosomal *mir*-15a [112, 113]. The myeloma BM-MSC derived exosomes also had higher oncogenic protein expression, cytokines and protein kinases relative to normal BMSC—derived exosomes.

Wang et al. also demonstrated that bone marrow stroma cell (BMSC) derived exosomes were crucial in communicating deleterious traits such as cell proliferation, migration and survival in multiple myeloma. The BMSC-derived exosomes induced resistance to bortezomib in myeloma cells in the 5T33 murine multiple myeloma model [130]. The study also validated the exchange of cytokines between BMSCs and myeloma cell derived exosomes by confocal microscopy. The cytokine array of the exosomes showed that BMSCs and MM cells can cross-talk and exchange cytokines through an exosome-mediated pathway. The BMSC-derived exosomes were found to be enriched with several chemotactic proteins such as stromal cell derived factor 1(SDF-1) and monocyte chemoattractant protein 1 (MCP-1) that effectively promoted in vitro myeloma cell migration. BMSC-exosomes also substantially increased the viability, proliferation, and survival capacity of multiple myeloma cells.

Through the same work, the authors also examined the effect of BMSC-exosomes on bortezomib-induced apoptosis. The caspase 9, 3 mediated apoptotic cascades resulting in PARP (poly-ADP ribose polymerase) cleavage is the mechanism of bortezomib action in MM and BMSC-derived exosomes inhibited this pathway and thereby protected the MM cell. RPMI8226 MM cell line was treated with BMSC-derived exosomes obtained from MM patient bone marrow samples to validate the induced resistance to bortezomib by BMSC-derived exosomes further. The BMSC-derived exosomes increased MM cell viability to 25% in the presence of bortezomib whereas only 9% of MM cells survived in the absence of bortezomib [130].

Harshman et al. analyzed the proteomic content of extracellular vesicles from myeloma cell lines MM 1S and U266 [55]. The study compared the proteome of MM 1S and U266 cell lysates to their respective extracellular vesicle population and found a significant overlap in proteomic content. This study used a novel label free approach to identify the relative abundance of proteins within and across these vesicles of distinct cell line origin to that of their parents. The study demonstrated that extracellular vesicles from two cell lines shared a common protein profile to a significant extent however, contained a small set of unique proteins with statistically distinct abundance. The study showed that MM.1S vesicles show increased abundance of Human Leukocyte Antigen (HLA) class II histocompatibility antigens when compared to the cell lysate. The role of this specific packaging was also previously described by Raposo et al. [109] where the MHC class II complexes stimulate T cells in vitro and the specific shedding of these molecules into vesicles enable myeloma cells to evade the recognition by CD8^**+**^ T cells supporting tumor survival.

### The diagnostic capabilities of microparticles in multiple myeloma

We first reported on the isolation, detection, morphology and numbers of systemic plasma cell derived MPs (CD41a^**−**^CD138^**+**^) in MM patients [76, 77]. Microparticles were isolated by differential high-speed centrifugation, as previously described by us [10] and validated by flow cytometry for typical characteristics of size and phosphatidylserine (PS) exposure.The isolated MPs displayed a spherical and smooth morphology with a mean size of 0.1–1 µm in diameter. Platelet-free plasma was used as the starting material to ensure that contamination by platelet-derived MPs in the final preparation was minimised. Microparticles arising from plasma cells were detected using anti-CD138-APC mAb. We observed greater CD138^+^ MP counts in MM patients relative to healthy subjects. Consistent with this, we observed greater CD138^+^ MP counts for patients in remission (CR and PR) and with progressive disease (PD) relative to healthy volunteers. In this study, we also identified 9 patients who were in complete remission (defined using the IMWG response criteria) at the time of analysis who had greater CD138^+^ MP counts relative to the rest of the cohort. All of the patients relapsed within a short time demonstrating the potential for CD138^+^ MP counts to predict the transition between remission and progressive disease before clinically used markers. We also reported on the prognostic potential for CD138^+^ MPs in predicting ‘risk of relapse’ in individual patients.

We have previously investigated the potential for MPs to serve as biomarkers in gauging therapeutic outcome and MDR in cancer. Our earlier work discovered that (i) MPs are spontaneously shed from tumor cells; (ii) they carry functional resistance proteins including P-gp, MRP1 and nucleic acids from their originating cell; and (iii) can confer MDR and increased metastatic capacity within cancer cell populations in a matter of hours [10, 47–49, 61, 64, 86, 104]. We thus explored the clinical relevance of P-gp^**+**^ MPs in MM patients.

We reported the presence of numerous MP subtypes when probing for the presence of P-gp on MPs, including the presence of a dual positive MP population (CD138^**−**^CD34^**+**^P-gp^**+**^) of ‘stem cell like’ origin, which was associated with an aggressive disease state [107]. The presence as well dominance of distinct MP subtypes demonstrate an evolving shift in the cell populations and phenotypes during disease progression following the treatment. Considering these studies, we conclude that CD138 that is synonymous with plasma cell burden cannot be considered a ‘static’ biomarker throughout the full course of disease; rather it appears relevant during responsive states and diminishes in aggressive disease. This has important implications in how we define the utility of biomarkers with respect to disease progression generally.

Extra-cellular vesicles are involved in the tumor biology by modifying the stroma supporting malignancy, inducing tumor drug resistance, and immune suppression. Moreover, in the context of hematological malignancies they could be potential biomarkers as a component of liquid biopsy [19]. Extra-cellular vesicles are richly present in biological fluids such as saliva, urine and blood. They have natural capacity to protect molecular cargo with potential of engineering them as therapeutic tools.

### Potential prognostic significance of Microparticle biomarkers in myeloma

As previously discussed by us in [76, 77], both the conventional agents as well as novel agents are substrates of ABC-transporter proteins. The clinical management of MM is heavily reliant on combination therapy and the wide range of structurally and functionally unrelated drug substrates impede successful treatment in MM. The novel agents, IMiDs (thalidomide derivatives) and proteasome inhibitors, have improved overall patient survival significantly, compared to the conventional agents [4, 85, 99, 129]. However, treating MM with novel agents also add significantly to the global healthcare financial burden [43]. Intriguingly, the agents used in combination therapy are substrates of ABC transporters {Mynott, 2021 #222}{Pinto, 2020 #237}. In this scenario, personalized approaches to treatment not only have the potential to improve survival but they are also cost-effective [76, 77].

### Syndecan 1 (CD138)

CD138 (Syndecan 1) is arguably the most unique marker for mature PCs and therefore important in detecting PCs in MM [97]. CD138 is a type 1 transmembrane heparan proteoglycan that facilitates interactions of PCs with extracellular matrix (ECM) and homing of PCs in the bone marrow [9, 50]. CD138 mediates MM cell interaction with type 1 collagen and cell to cell adhesion along with a regulated interaction with growth factors in the bone marrow microenvironment [116]. However, in the malignant state CD138 becomes a significant player in MM progression with its dynamic capacity to convert into a soluble effector molecule [9, 116].

Structurally, CD138 possesses a highly conserved cytoplasmic region at the –COOH terminus and an extracellular domain (ectodomain) at the –NH2 terminus bearing heparan sulphate (HS) or chondroitin sulphate (CS) chains [13]. The short (28–34 amino acids) cytoplasmic region consists of a single variable segment flanked by two constant regions and adhere to intracellular ligands such as kinases or structural proteins  [13, 14, 123]. The ectodomain acts as a classical co-receptor for growth factors and a range of biomolecules such as cytokines, proteases and cell adhesion molecules for PCs [9, 50, 95]. In an unstimulated state, syndecan 1 or CD138 binds to ECM components, adhesion molecules, proteins involved in lipid metabolism, proteinases and proteinase inhibitors via the HS and CS chains (Merton [13, 14]. The interaction of the ectodomain with growth factors are further influenced by size and heterogeneity of HS and CS chains on CD138 ectodomain which in turn affects MM cell behaviour [116].

In the context of myeloma; clonotypic B cells which share same variable diversity joining (VDJ) arrangements and/or MM stem cells known as ‘side population’ have been shown to play a role in MM relapse and its incurability [4, 84, 89]. This should be considered in alignment with the ABC- transporters induced MDR in MM as the ‘side population’ cells typically lack CD138, and display stem cell characteristics referred as clonogenic cells [54]. Clonogenic cells also classically overexpress the ABC transporters on their surface with their presence evident by low intracellular accumulation of Hoechst 33,342 dye [84] and is characterised phenotypically different to mature PCs. Clonogenic cells express early B cell markers on their surface and possess self-renewal capacity [4]. Failure of chemotherapeutics to eradicate the clonogenic cells or ‘side population’ is one of the major reasons for unsuccessful therapeutic outcome in MM [4, 60]. Therefore, frequent monitoring of clonogenic cell characteristics such as MDR markers is necessary to proactively minimize relapse [35, 74, 89].

Further, the heparan sulphate bearing ectodomain of CD138 is shed as a whole by proteolytic sheddases (also secretases or convertases) as a response to physiological stimuli such as chemotactic peptides, cytokines, calcium ionophores [123]. The cleaving occurs at the juxtamembrane domain and is specifically thought to be at a dibasic region (Lys-Arg) closer to the outer leaf of the plasma membrane [123] (which, interestingly makes them readily a cargo of MPs during plasma membrane remodelling in general). The resultant soluble effector molecule maintains the binding capacity of their surface predecessors by means of intact HS or CS chains [9, 39]. Numerous developmental and or pathophysiological events like wound healing and cancer biology are indeed affected by ectodomain shedding of CD138 triggering various intracellular pathways [39]. The proteolytic cleavage is closely associated with the outer surface of the cell and is regulated by tissue inhibitor of metalloproteinase-3 (TIMP-3). Moreover, agents triggering cellular stress response via receptor activation (thrombin, plasmin) can accelerate the ectodomain cleavage and the mechanisms involved in accelerated, to that of constitutive shedding are distinct [39]. Fritzgerald et al. [39] demonstrated the existence of distinct mechanism as TIMP-3 and hydroxamate inhibit accelerated shedding whereas constitutive shedding is unaffected by TIMP-3 and requires approximately ten-fold higher hydroxamate for inhibition [39].

In multiple myeloma, the shed CD138 accumulates in the fibrotic region of the bone marrow as well as systemically in the circulatory system [9, 58], Carina Seidel, Anders Sundan, et al., 2000). In the context of biomarker status, systemically shed CD138 is associated with a negative prognosis in MM [33, 118, 119]. Specifically, systemic CD138 was shown to be an independent prognostic indicator and correlated to β_2_M, creatinine, serum, urinary M protein and S-IL6-R in the Nordic myeloma group study [118, 119]. Another study showed higher soluble CD138 was correlated with tumor mass in MM [33, 118, 119]. CD138 also has a role in promoting tumor vascularisation, thereby stimulating angiogenesis and supporting tumor growth as well as dissemination in MM [9, 18, 73, 81, 83]. Khotskaya et al. showed that silencing CD138 by RNA interference in vitro*,* resulted in cell death in MM cells (human MM cell lines, RPMI 8226, CAG (cell line established from the bone marrow aspirate of an MM patient), indicating CD138’s role in MM pathogenesis [73]. In the same study, CD138 was silenced in a mouse xenograft model and the cells also showed low levels of vascular endothelial growth factor (VEGF) expression. Subsequently, fewer, smaller and less invasive subcutaneous tumors were formed [73].

Seidel et al*.* demonstrated that hepatocyte growth factor (HGF) produced by MM cells is believed to have a role in bone resorption in the microenvironment and is associated with negative prognosis. The soluble CD138 effector molecule and HGF formed a complex, subsequently increasing HGF’s half-life in BM (Carina Seidel, Magne Børset, et al., 2000). This demonstrates the supplementary role of shed syndecan 1 in MM pathology by influencing factors like cytokines in the BM microenvironment.

### Phosphatidyl Serine (PS)

PS is a ubiquitous marker of MPs arising from loss of phospholipid asymmetry during MP biogenesis [114]. However, it is also known that PS is not an exclusive marker with variable expression within the MP population [69, 92]. PS is also emerging as an important mediator in extracellular vesicle biology. A recent study showed that PS in hypoxia induced mesenchymal stem cell derived microvesicles was crucial in the internalisation of vesicles into human umbilical cord endothelial cells (HUVECs) [131] suggestive of a role in supporting angiogenesis. We observed significantly elevated numbers of PS^**+**^ MPs across all MP subtypes and elevated levels were observed specifically across active disease states. The significance of the increased PS^**+**^ MP event in myeloma is currently unknown and may be linked to the dissemination of malignant cells to extramedullary sites during disease progression [131].

### CD34

CD34 is a single-pass transmembrane protein belonging to the CD34 family of sialomucins and specific for cell surface marker of immature or precursor cells [117]. CD34 is expressed in hematopoietic precursor and mature endothelial cells in human, but it is not thought to be expressed in myeloma cells or tissue. The function of CD34 is not well defined however studies suggest that it participates in the cellular adhesion of endothelial cells [110]. In myeloma clinical setting, CD34 + stem cells with minimal contamination from clonotypic cells are used in hematopoietic stem cell transplantation as it is believed that myeloma cells lack CD34 [52]. However, Kuranda et al. demonstrated that MM patients do carry a subpopulation of CD34^+^ positive and could limit the effectiveness of CD34 selection during ASCT purging [78]. This is of particular interest as some patients respond very poorly to transplant and results in poor therapeutic outcome in aggressive disease [107].

Similarly, in myeloma, as discussed above, heterogeneous clonal mixtures with an altered dominance in clones constitute one of the complicating factors in clinical management. Our research in this particular space indicate that MPs provide a very sensitive picture of the “evolving shift” in the dominance of cancer progenitor cells with disease progression. The presence of ‘stem cell like origin’ (CD34) MPs in aggressive disease and the diminishing presence of CD138 on MP surface in progressive disease indicated the dynamic shift during the course of therapy in our patient cohort. This is corroborated with reduced PS exposure on CD138** + **subtype of MPs and the lack of co-localization of P-gp and CD138 in aggressive disease states.

## Conclusion

This review has examined current systemic markers in multiple myeloma in the context of disease biology and their limitations in giving substantial information in optimising desirable therapeutic outcome in patients. We discussed the factors that can impact on patient responsiveness and the need for individualised approaches to maximise treatment efficacy and survival. Therapeutic response in multiple myeloma is unpredictable with the evolution of drug resistance during the course of therapy, and there is a need for a systemic clinical tool which can gauge the evolution of MDR, predict risk of relapse and monitor disease progression routinely in the clinical management of myeloma. Existing clinical tools are limited as they provide an indirect measurement of tumor burden, cannot directly measure resistance protein expression routinely or non-invasively and cannot capture the patchy, multi-site clonal infiltrates associated with myeloma.

Our prior studies have shown that MPs are effective vectors in the transfer of MDR proteins in vitro and in vivo. The presence of MDR proteins within the vesicle cargo makes them potential biomarkers with prognostic potential for gauging the development of MDR in myeloma.

Indeed, our recent clinical studies have shown that the number, phenotypes of MPs in myeloma are indicative of patient response state, the emergence of MDR and disease progression [107]. The demonstration that the emergence of P-gp^**+**^ MDR in myeloma can be detected and monitored serially by analysing MPs in patient blood samples makes a significant contribution to achieving this goal. We also discuss the need to reassess the utility of defined biomarkers during disease progression given our observations for the diminishing presence of CD138, the classic myeloma cell marker in aggressive and/ or progressive stages of disease in myeloma. The existence and dominance of MP subtypes reflect the evolving and ever-changing dominant cell populations during the course of disease and in response to treatment. Taken together, in defining biomarkers, careful consideration should be given in this context.

As widely documented in the literature; MPs are detected systemically in healthy individuals; nonetheless higher levels are indicative of cellular activation across several pathologies [37, 44, 127]. Hence, the detection of circulating cancer-derived MPs from different cancers, has defined them as promising “surrogate” markers in compartmentalised malignancies (i.e. brain and bone) [45, 66, 121, 128]. Surface phenotyping using flow cytometric technique is the gold-standard applied in MP analysis as they display various cell surface markers denoting their cellular origin [10, 93]. This aligns well with the routine flow cytometric applications used in routine hematology and in the current myeloma clinical setting. This enables a seamless integration of this approach to current cinical setting. In combination with existing clinical tools, this integration would provide for a thorough and systematic assessment of the complete disease landscape in individual myeloma patients. A routine assessment and monitoring patients for the ‘risk of relapse’ prior to clinical manifestation has the potential to tailor treatment regimens to patient characteristics and see increase in progression free survival.

As discussed, there is currently no cure for multiple myeloma, making the clinical management and ‘control’ of the condition of utmost importance for optimising patient quality of life. The approaches to treatment myeloma are generally divided into; ‘cure’ where hard-hitting chemotherapy is adopted and secondly, ‘control’ where the emphasis is on the maintaining quality of life. The numbers and molecular profiling of MPs can potentially assist with both. Furthermore, the individualized approach in therapeutic management of myeloma addresses the vast inter-individual variability limiting current generalised approaches. As of now, optimization of isolation methods, characterization of specific identity and origin of extracellular vesicles from biological fluids is still in progress. There is also insufficient information about their biology, activity in pathology spectrum and in general, health. These aspects remain open for further exploration and standardization [19].

In conclusion, we have provided evidence that MDR myeloma patients can be detected and serially monitored by analyzing MPs in blood samples in the context of a ‘liquid biopsy’ [107]. Our results indicate the presence of markers of MDR on MPs of stem cell-like origin. Stem cells are a reservoir of ABC Transporters, the levels of which appear to correspond to treatment outcome. This information has significant implications in the design of effective treatment strategies, including targeted approaches against distinct cell clones with discrete phenotypes. The shifting dominance of these signatures present at various times must be considered during the design of treatment interventions.

The work we present here depicts a personalized approach with prognostic potential in determining the evolution of MDR in MM, whereby the development of MDR can be serially and minimally-invasively monitored by analysing circulating MPs in the context of a with personalized prognostic capacity for determining the evolution of disease progression provides a relevant addition to the current repertoire of prognostic clinical tools. The ability to continuously monitor patients during treatment would allow for improved patient survival as alternative treatments can be initiated promptly to prevent re-occurrence of significant tumor burden. This would certainly be useful in cases of non-secretory myeloma, which lack the classic manifestation of elevated Mprotein levels. These new insights into the molecular mechanisms contributing to disease progression, MDR and treatment failure in myeloma, assist to identify key biomarkers, introduce new approaches focussed for disease state management in multiple myeloma.

## Data Availability

NA.

## Abbreviations

MM: Multiple myeloma
MP: Microparticles
PCs: Plasma cells
MDR: Multidrug resistance
ABC transporters: ATP- binding cassette transpoters
IMiDs: Immunomodulatoryimid drugs
EV: Extracellular Vesicle
MGUS: Monoclonal Gammopathy of Undetrmined Significance
BM: Bone Marrow
PC: Plasma cell
TK: Thymidine kinase
β2M: Beta-2-microglobulin
PARP: Poly ADP Ribose Polymerase
HLA: Humal Leukocyte Antigen
BMSC: Bone Marrow Stromal Cell
BM-MSC: Bone Marrow Mesenchymal Stromal Cell
P-gp: P-glycoprotein
IL-6: Interleukin-6
CRP: C-Reactive Protein
s-IL6-R: Soluble IL-6 Receptor
LDH: Lactate dehydogenase
PICP: Type 1 carboxy terminal propeptide, type 1 collagen biosynthesis marker
ICTP: Type 1 carboxy terminal cross-linked telopeptide
NGS: Next Generation Sequencing
MRP1: Multidrug Resistance associated Protein 1
SDF-1: Stromal cell Derived Factor
1MCP-1: Monocyte Chemosttractant Protein -1
PS: Phosphatidyl Serine
MRD: Minimal Residual Disease
VEGF: Vascular Endothelial Growth Factor
ECM: Extra Cellular Matrix
CR: Complete Remission
PR: Partial Remission
PD: Progressive disease
CS: Chondroitin Sulphate
HS: Heparan Sulphate
TIMP-3: Tissue inhibitor of metalloproteinase-3
HGF: Hepatocyte Growth Factor
HUVECs: human umbilical cord endothelial cells
